# Improving the accuracy of genomic evaluation for linear body measurement traits using single-step genomic best linear unbiased prediction in Hanwoo beef cattle

**DOI:** 10.1186/s12863-020-00928-1

**Published:** 2020-12-02

**Authors:** Masoumeh Naserkheil, Deuk Hwan Lee, Hossein Mehrban

**Affiliations:** 1 Department of Animal Science, University College of Agriculture and Natural Resources, University of Tehran, P.O. Box: 4111, Karaj, 77871-31587 Iran; 2 Department of Animal Life and Environment Sciences, Hankyong National University, Jungang-ro 327, Anseong-si, Gyeonggi-do South Korea; 3 Department of Animal Science, Shahrekord University, P.O. Box: 115, Shahrekord, 88186-34141 Iran

**Keywords:** Body measurement traits, Single-step, Genomic prediction, Hanwoo cattle

## Abstract

**Background:**

Recently, there has been a growing interest in the genetic improvement of body measurement traits in farm animals. They are widely used as predictors of performance, longevity, and production traits, and it is worthwhile to investigate the prediction accuracies of genomic selection for these traits. In genomic prediction, the single-step genomic best linear unbiased prediction (ssGBLUP) method allows the inclusion of information from genotyped and non-genotyped relatives in the analysis. Hence, we aimed to compare the prediction accuracy obtained from a pedigree-based BLUP only on genotyped animals (PBLUP-G), a traditional pedigree-based BLUP (PBLUP), a genomic BLUP (GBLUP), and a single-step genomic BLUP (ssGBLUP) method for the following 10 body measurement traits at yearling age of Hanwoo cattle: body height (BH), body length (BL), chest depth (CD), chest girth (CG), chest width (CW), hip height (HH), hip width (HW), rump length (RL), rump width (RW), and thurl width (TW). The data set comprised 13,067 phenotypic records for body measurement traits and 1523 genotyped animals with 34,460 single-nucleotide polymorphisms. The accuracy for each trait and model was estimated only for genotyped animals using five-fold cross-validations.

**Results:**

The accuracies ranged from 0.02 to 0.19, 0.22 to 0.42, 0.21 to 0.44, and from 0.36 to 0.55 as assessed using the PBLUP-G, PBLUP, GBLUP, and ssGBLUP methods, respectively. The average predictive accuracies across traits were 0.13 for PBLUP-G, 0.34 for PBLUP, 0.33 for GBLUP, and 0.45 for ssGBLUP methods. Our results demonstrated that averaged across all traits, ssGBLUP outperformed PBLUP and GBLUP by 33 and 43%, respectively, in terms of prediction accuracy. Moreover, the least root of mean square error was obtained by ssGBLUP method.

**Conclusions:**

Our findings suggest that considering the ssGBLUP model may be a promising way to ensure acceptable accuracy of predictions for body measurement traits, especially for improving the prediction accuracy of selection candidates in ongoing Hanwoo breeding programs.

**Supplementary Information:**

The online version contains supplementary material available at 10.1186/s12863-020-00928-1.

## Background

Improving meat production in beef cattle is an important breeding goal throughout the world because it has a considerable effect on the profitability of the beef industry. Linear body measurement traits are important economic traits in beef cattle, as they provide useful information for understanding the growth and frame size of the animals. The relationship between the linear type traits and economically important traits, such as reproductive traits [1, 2], longevity [3, 4], lifetime production efficiency [5], and growth traits [6, 7], have been extensively researched in both dairy and beef cattle.

Hanwoo cattle are unique to Korea and popular for meat owing to their rapid growth and high quality beef. Body measurements have become routinely collected traits over the last three decades in this breed, which provide valuable resources to study the complete growing period [8]. Moreover, these traits have been proposed as indirect selection criteria for the genetic improvement of meat production in beef cattle [9, 10], and can be harnessed to accelerate the breeding progress. Improving the accuracy of selection for body measurement traits will benefit the beef cattle industry; consequently, these traits are often included in multi-trait genetic evaluations as predictors of performance in beef cattle [11, 12]. To this end, the application of genomic selection could be a promising tool to improve the accuracy of estimation of breeding values of body measurement traits. It refers to selection based on genomic estimated breeding values (GEBV) using genome-wide marker information [13] instead of the traditional selection which uses pedigree-based BLUP [14]. Several statistical methods were developed to predict GEBV from 2001 onwards, among which the genomic best linear unbiased prediction (GBLUP) models and Bayesian variable selection or variable shrinkage models have been widely used [13, 15, 16]. The main differences between these models are the assumptions of the distribution of single nucleotide polymorphism (SNP) effects. Nonetheless, the GBLUP method has become a popular approach for practical genomic evaluations because most traits in livestock species have polygenic nature [17–20], and also because of its simpler and lower computational demand than other methods [21]. A decade ago, a method based on the GBLUP framework was proposed by Misztal et al. [22], termed the single-step genomic best linear unbiased prediction (ssGBLUP), which uses simultaneously all pedigree, genotypic and phenotypic information from both genotyped and non-genotyped individuals. In this method, the pedigree-based numerator relationship matrix (**A**) and relationship matrix based on genomic information (**G**) are combined into a single matrix (**H)** [22, 23]. The use of ssGBLUP increases the accuracy of genomic prediction compared to the methods using only genotyped individuals [24]. In this line, previous studies have demonstrated that the accuracy of genomic evaluation in many species could be increased by using ssGBLUP compared with pedigree-based BLUP or genomic BLUP [25–32]. Besides, the literature on prediction of the breeding values of linear body measurement traits using genomic evaluations in field data of dairy cattle [25, 33, 34], beef cattle [35–37], goats [38–40], sheep [41], and pig [42] have been previously reported.

Since there is sufficient pedigree information available in Hanwoo cattle [31, 32], it is expected that the use of ssGBLUP can be influenced to improve genomic prediction accuracy for body measurement traits. Nonetheless, these traits have not yet been investigated in breeding programs for this breed. Therefore, the aim of this study was to evaluate the accuracy of breeding values for linear body measurement traits using conventional BLUP only on genotyped animals (PBLUP-G), conventional BLUP with all animals, GBLUP, and ssGBLUP methods, which provide valuable insights into the application of genomic selection for the studied traits in Hanwoo beef cattle.

## Results

### Descriptive statistics and estimates of variance components

The number of animals with records, means, minimum, maximum, standard deviations, and phenotypic coefficient of variation for the 10 body measurement traits are shown in Table 1. The mean values of body measurement traits ranged from 21.38 to 165.95 with standard deviation between 2.71 and 9.48. Variance components and heritability estimates for the studied traits are presented in Table 2. The range of heritability for the 10 traits was between 0.11 and 0.40. Among all investigated traits, the estimated heritability of BH was the highest (0.40) and the lowest was for CW and HW (0.11). The standard error for all heritability estimates was less than 0.03 (Table 2).

**Table 1:** Summary statistics for linear body measurement traits used to estimate variance components in Hanwoo cattle

Trait ^a^ (Unit)	No. records	Mean (SE)^b^	Min.	Max.	SD	CV%
BH (cm)	13,066	119.27 (0.04)	84	138	5.06	4.24
BL (cm)	13,024	132.69 (0.06)	85	150	7.18	5.41
CD (cm)	13,061	61.38 (0.03)	27	77	3.40	5.54
CG (cm)	13,066	165.95 (0.08)	101	202	9.48	5.72
CW (cm)	13,065	37.33 (0.04)	20	67	4.29	11.49
HH (cm)	13,064	121.64 (0.04)	84	141	5.00	4.11
HW (cm)	13,067	21.38 (0.02)	8	40	2.71	12.66
RL (cm)	13,065	44.41 (0.03)	24	63	3.49	7.85
RW (cm)	13,066	39.37 (0.03)	21	58	3.11	7.90
TW (cm)	13,065	37.19 (0.03)	4	68	3.10	8.33

^a^ *BH* Body height, *BL* Body length, *CD* Chest depth, *CG* Chest girth, *CW* Chest width, *HH* Hip height, *HW* Hip width, *RL* Rump length, *RW* Rump width, *TW* Thurl width, ^b^ *SE* Standard error

**Table 2:** Pedigree-based BLUP variance component estimations and heritability of linear body measurement traits in Hanwoo cattle

Trait ^a^	Additive genetic variance (SE)^b^	Residual variance (SE)	Phenotypic variance (SE)	Heritability (SE)
BH	5.61 (0.47)	8.40 (0.34)	14.01 (0.22)	0.40 (0.03)
BL	4.63 (0.56)	20.17 (0.49)	24.80 (0.34)	0.19 (0.02)
CD	1.13 (0.13)	4.61 (0.12)	5.74 (0.08)	0.20 (0.02)
CG	5.89 (0.77)	29.48 (0.69)	35.37 (0.48)	0.17 (0.02)
CW	0.87 (0.14)	6.74 (0.14)	7.61 (0.01)	0.11 (0.02)
HH	5.55 (0.46)	8.60 (0.34)	14.15 (0.22)	0.39 (0.03)
HW	0.28 (0.04)	2.30 (0.04)	2.59 (0.03)	0.11 (0.02)
RL	0.91 (0.12)	4.52 (0.10)	5.42 (0.07)	0.17 (0.02)
RW	0.80 (0.10)	4.14 (0.09)	4.94 (0.07)	0.16 (0.02)
TW	0.73 (0.10)	4.82 (0.10)	5.55 (0.07)	0.13 (0.02)

^a^ *BH* Body height, *BL* Body length, *CD* Chest depth, *CG* Chest girth, *CW* Chest width, *HH* Hip height, *HW* Hip width, *RL* Rump length, *RW* Rump width, *TW* Thurl width, ^b^ *SE* Standard error

### Comparison of accuracy and bias for the four models

**Fig. 1:**
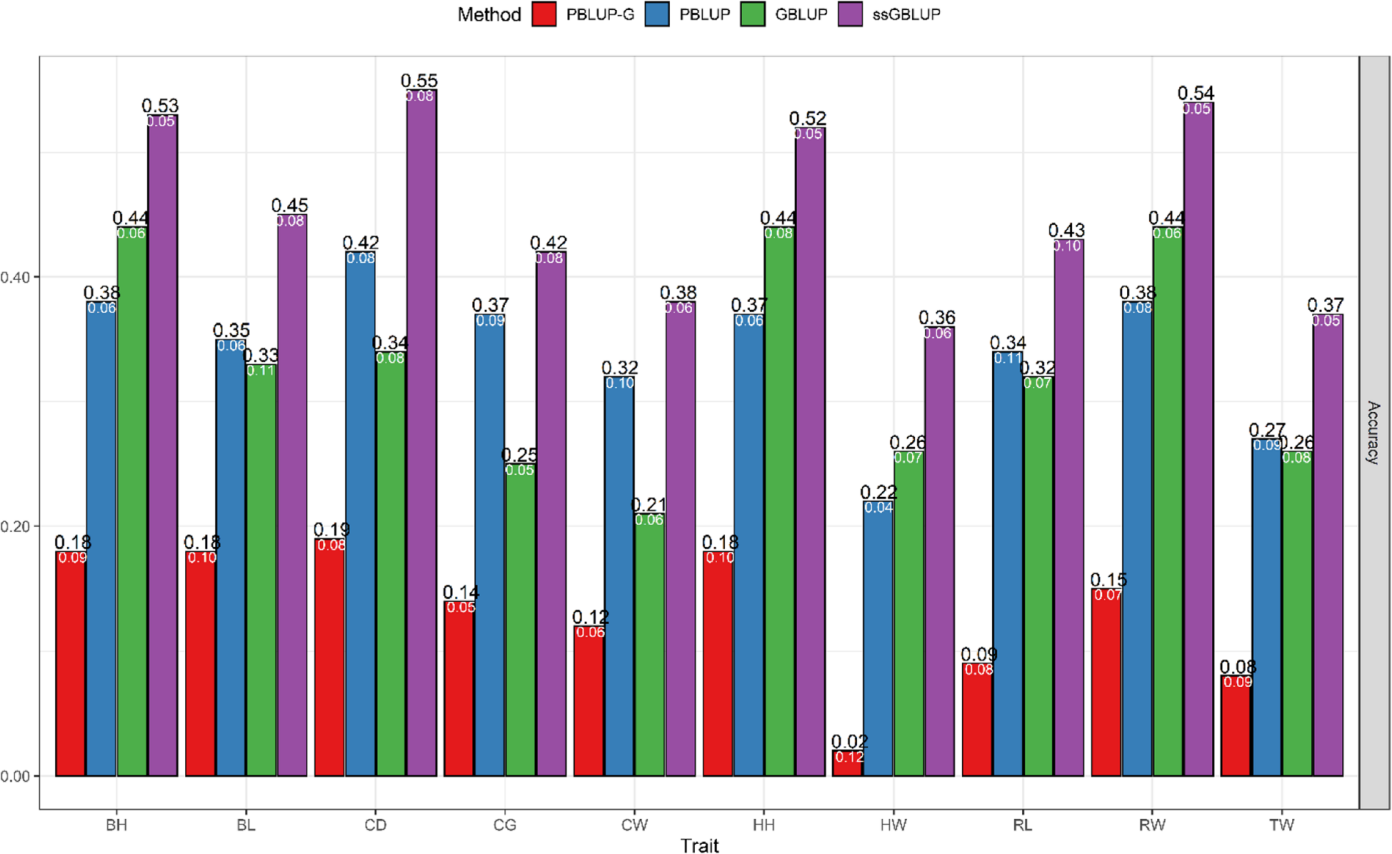
Accuracy of breeding values obtained using PBLUP-G, PBLUP, GBLUP, and ssGBLUP methods. The means and standard errors for body height (BH), body length (BL), chest depth (CD), chest girth (CG), chest width (CW), hip height (HH), hip width (HW), rump length (RL), rump width (RW), and thigh width (TW) in Hanwoo cattle. The white numbers represent standard error (SE). PBLUP-G, pedigree-based best linear unbiased prediction only on genotyped animals; PBLUP, pedigree-based best linear unbiased prediction with all animals; GBLUP, genomic best linear unbiased prediction; ssGBLUP, single-step genomic best linear unbiased prediction

The predictive accuracies for the 10 traits obtained using PBLUP-G, PBLUP, GBLUP, and ssGBLUP methods are shown in Fig. 1. The accuracies determined using PBLUP-G, PBLUP, GBLUP, and ssGBLUP methods ranged from 0.02 to 0.19, 0.22 to 0.42, 0.21 to 0.44, and 0.36 to 0.55, respectively. The average predictive accuracies across traits were 0.13 for PBLUP-G, 0.34 for PBLUP, 0.33 for GBLUP, and 0.45 for ssGBLUP methods (Fig. 1). The results showed that the average magnitude of improved accuracy from PBLUP to ssGBLUP was 33%, followed by changing the model from GBLUP to ssGBLUP was 43% for the 10 traits. Using data from only genotyped animals, prediction accuracies of the GBLUP method were considerably higher than those of the PBLUP-G method for all traits, whereas the average accuracies using GBLUP were slightly lower than PBLUP method. The results also indicated that when phenotypic data from only genotyped individuals were used, prediction accuracies of the PBLUP-G method were considerably lower than those of other methods for all traits. Furthermore, the ssGBLUP method provided higher accuracies of prediction than both PBLUP and PBLUP-G methods in all body measurement traits. The highest and lowest improvement of accuracy in ssGBLUP over PBLUP was obtained for HW (61%) and CG (14%) traits, respectively.

The scale of predictions is an important factor that determines the use of estimated breeding values for genetic evaluation. The regression coefficient of adjusted phenotypes was calculated as a measure of prediction bias (Table 3). The regression coefficients of prediction determined using PBLUP-G, PBLUP, GBLUP, and ssGBLUP methods ranged from 0.01 to 0.85, 0.69 to 1.05, 0.95 to 1.43, and 0.96 to 1.24, respectively, for all traits. The average regression coefficients across the traits were 0.54, 0.96, 1.13, and 1.12 using PBLUP-G, PBLUP, GBLUP, and ssGBLUP methods, respectively. There was no clear trend demonstrating that a model was better than the others regarding unbiased predictions for most traits. In addition, the average absolute deviation of regression coefficients from 1.0 was 0.46, 0.07, 0.15, and 0.13 for PBLUP-G, PBLUP, GBLUP, and ssGBLUP, respectively, indicating that the PBLUP method was the least biased, whereas the predictions from GBLUP and ssGBLUP methods tended to be slightly deflated for most traits (Table 3). However, RMSEs obtained using the ssGBLUP method were lower than those obtained by the PBLUP-G, PBLUP and GBLUP methods for all traits of interest (Table 4).

**Table 3:** Regression coefficients of the adjusted phenotypes of EBV/GEBV for PBLUP-G, PBLUP, GBLUP, and ssGBLUP methods

Trait ^a^	PBLUP-G	PBLUP	GBLUP	ssGBLUP
BH	0.56 (0.30)	1.04 (0.12)	1.17 (0.10)	1.19 (0.08)
BL	0.85 (0.55)	1.02 (0.10)	1.12 (0.39)	1.11 (0.14)
CD	0.76 (0.30)	1.05 (0.17)	1.22 (0.28)	1.24 (0.17)
CG	0.72 (0.24)	1.05 (0.28)	0.96 (0.22)	1.05 (0.19)
CW	0.71 (0.24)	0.99 (0.32)	0.99 (0.27)	1.07 (0.14)
HH	0.55 (0.29)	1.00 (0.12)	1.12 (0.14)	1.18 (0.07)
HW	0.01 (0.54)	0.69 (0.15)	0.95 (0.24)	0.96 (0.19)
RL	0.36 (0.37)	1.01 (0.30)	1.22 (0.25)	1.17 (0.25)
RW	0.62 (0.27)	0.99 (0.17)	1.43 (0.18)	1.24 (0.07)
TW	0.32 (0.55)	0.81 (0.25)	1.09 (0.37)	1.03 (0.17)

^a^ *BH* Body height, *BL* Body length, *CD* Chest depth, *CG* Chest girth, *CW* Chest width, *HH* Hip height, *HW* Hip width, *RL* Rump length, *RW* Rump width, *TW* Thurl width

**Table 4:** Root of mean square error (RMSE) of EBV/GEBV for PBLUP-G, PBLUP, GBLUP, and ssGBLUP methods

Trait ^a^	PBLUP-G	PBLUP	GBLUP	ssGBLUP
BH	4.03 (0.06)	3.94 (0.08)	3.89 (0.05)	3.83 (0.08)
BL	5.42 (0.12)	5.37 (0.13)	5.39 (0.13)	5.33 (0.14)
CD	2.32 (0.08)	2.30 (0.09)	2.31 (0.08)	2.27 (0.09)
CG	5.92 (0.19)	5.87 (0.18)	5.94 (0.16)	5.86 (0.18)
CW	2.98 (0.07)	2.96 (0.07)	2.98 (0.06)	2.95 (0.07)
HH	4.12 (0.07)	4.03 (0.10)	3.99 (0.07)	3.92 (0.10)
HW	1.68 (0.10)	1.68 (0.10)	1.68 (0.10)	1.67 (0.10)
RL	2.60 (0.03)	2.57 (0.03)	2.58 (0.03)	2.55 (0.03)
RW	2.28 (0.03)	2.25 (0.04)	2.25 (0.03)	2.22 (0.04)
TW	2.47 (0.04)	2.45 (0.04)	2.46 (0.05)	2.44 (0.04)

^a^ *BH* Body height, *BL* Body length, *CD* Chest depth, *CG* Chest girth, *CW* Chest width, *HH* Hip height, *HW* Hip width, *RL* Rump length, *RW* Rump width, *TW* Thurl width

## Discussion

The heritability estimates for the 10 body measurement traits in this study were low to relatively high, ranging from 0.11 to 0.40. In the same breed, Choy et al. [43] reported heritability estimates for BL (0.23), CD (0.28), CG (0.27), CW (0.21), HW (0.20), and RW (0.26) at 12 months of age, which were somewhat higher than those seen in our study. These discrepancies could be due to the difference in the numbers of animals measured (approximately 32% more in this study than their study on Hanwoo cattle) and statistical models used for variance component estimations. The estimated heritability of HH was consistent with the study by Zhang et al. [44], where the estimates of heritability for HH at yearling age was 0.38 in Chinese Holstein. The estimate of the heritability of CW in our study was lower than those reported previously for Holstein in the literature [33, 34]. Moreover, our heritability estimates for CD, CW, HW, and TW are within the ranges of the heritability estimates reported by Doyle et al. [45] in Angus, Charolais, Hereford, Limousin, and Simmental cattle.

In this study, we investigated the accuracy of the breeding values for linear body measurement traits using four models, pedigree-based BLUP only on genotyped animals (PBLUP-G), pedigree-based BLUP with all animals (PBLUP), genomic BLUP (GBLUP), and single-step genomic BLUP (ssGBLUP) in Hanwoo beef cattle. Accuracies of obtained breeding values from these models were compared. To maximize the profitability of the beef cattle industry, selection for economically important traits is desirable. Estimations of breeding values for body measurement traits are most important because they are widely used as predictors of growth [7], meat production and longevity [3] traits in the beef cattle industry. Several previous studies on Hanwoo cattle [31, 32] and on other beef cattle breeds [28, 29] have shown that the ssGBLUP outperformed GBLUP or pedigree-based BLUP methods for the prediction of breeding values in carcass and growth traits.

According to our results, the average predictive accuracies of 10 traits obtained using the ssGBLUP model were approximately 33% higher than those obtained using the PBLUP model, which ranged from 14% for CG to 61% for HW trait. The explanation of the observed gain in the accuracy is that the simultaneous use of pedigree, phenotypic, and genomic information in the single-step method provides additional information for estimating breeding values compared to traditional pedigree-based models, which are based on capturing the variation in Mendelian sampling [27]. Moreover, the average gain in accuracy across traits using the ssGBLUP method was approximately 43% higher relative to GBLUP method that only uses data from genotyped animals for all traits, which may reflect the fact that the use of additional phenotypic information from including non-genotyped animals along with a relatively deep pedigree, was available on Hanwoo cattle. On average, the GBLUP method was slightly less accurate than the PBLUP method, which could be attributable to a small number of genotyped reference animals. Another reason is that the PBLUP method utilizes all phenotypic data and pedigree information from all generations to predict breeding values, whereas the GBLUP model uses information only from genotyped animals in the current generation. Based on our results, the GBLUP method considerably outperformed the PBLUP-G model with the same phenotypic data for all traits. Similarly, Lee et al. [31] indicated that methods using genomic information from only genotyped animals performed better than PBLUP and PBLUP-G for carcass traits in Hanwoo cattle. It is important to note, however, that the accuracy of GBLUP using only genotyped animals available on the current generation was 96% of the prediction accuracy of pedigree-based BLUP over all generations. Therefore, it can be argued that the GBLUP method is highly beneficial when pedigree information is unavailable. For instance, in other species such as fish [46] and wild species [47], in which the information on the relationship is not available or can be difficult to keep track of, the GBLUP method can be a useful strategy for improving the accuracy of prediction.

Consistent with our results, Song et al. [42] achieved a lower accuracy with GBLUP method than PBLUP and ssGBLUP methods for all seven body measurement traits in pigs. They also demonstrated that on average, the accuracies of genomic prediction using the ssGBLUP method were higher by 86 and 1% than those using GBLUP and PBLUP, respectively. In another study, Lourenco et al. [48] also showed that GBLUP method was less accurate than PBLUP method for fat percentage trait in all parities using a small number of genotyped animals in the dairy population. Similarly, Abo-Ismail et al. [34] reported that the average reliabilities of EBVs was higher than the average reliabilities of direct genomic breeding values (DGV) using different SNP sets for body conformation traits in the validation population of Holstein cattle.

Some studies have been undertaken to investigate the prediction accuracy of evaluations using the ssGBLUP method for linear type and body measurement traits on different animals such as dairy cattle [25, 33], dairy goat [38–40], dairy sheep [41], and pig [42], which highlights that the ssGBLUP method is as accurate as or more accurate than either the PBLUP or GBLUP method for the traits of interest. For instance, Tsuruta et al. [25] exhibited that ssGBLUP method was more accurate than the PBLUP method for 18 linear-type traits in Holsteins. Their results showed that the average reliabilities of 18 traits from the single-trait ssGBLUP model were 86% higher than those from the single-trait BLUP model, and the gain in the reliability of breeding values determined using the multi-trait ssGBLUP model was, on average, 84% compared with that obtained using the multi-trait BLUP for all traits. Similarly, the superiority of ssGBLUP over PBLUP was reported in the estimation of the breeding values for 20 linear-type traits of Holstein cows by Zavadilová et al. [33], who found that the average correlation between the post-progeny test EBV and parent average (0.30) was lower than the average correlation between the post-progeny test EBV and the predicted GEBV (0.40).

Previously, the performance of ssGBLUP has been reported to be better than either the pedigree-based BLUP or GBLUP for milk production traits, udder type traits, and somatic cell scores in French dairy goats and delivered a 61 to 96% gain in the accuracy of genomic prediction for udder type traits [38, 39]. Furthermore, Oget et al. [41] showed that ssGBLUP performed more accurately than pedigree-based BLUP for type traits consisting of teat angle (15.84%) and udder depth (26.07%) in Lacaune dairy sheep.

Overall, our findings indicate that ssGBLUP generally generated higher prediction accuracy than the other three methods for body measurement traits in Hanwoo cattle, which could be implemented in practical breeding programs.

## Conclusions

This study aimed to improve the accuracy of genomic prediction through the incorporation of the information of genotyped and non-genotyped animals into a genetic evaluation for body measurement traits in Hanwoo beef cattle. Four methods were also compared, PBLUP-G, PBLUP, GBLUP, and ssGBLUP in terms of accuracy. Our results demonstrate that the ssGBLUP provides a more accurate prediction than both traditional BLUP (PBLUP-G and PBLUP) and GBLUP for all the studied traits. It is worth noting that the ssGBLUP yielded on average 43% higher accuracy than GBLUP and 33% higher accuracy than PBLUP on body measurement traits. Therefore, the ssGBLUP can be considered as an alternative for effectively improving the prediction accuracy of selection candidates in ongoing Hanwoo breeding schemes.

## Methods

### Pedigree and phenotypic data

The dataset used in this study was provided by the Hanwoo Improvement Center of the National Agricultural Cooperative Federation and included 8452 bulls and 4615 steers born between 1989 and 2015. The pedigree consisted of 50,220 animals, which were traced back to 11 generations. The phenotypic data of body measurement traits were recorded in centimeters as continuous traits at the age of 12 months. The measured traits included body height (BH), body length (BL), chest depth (CD), chest girth (CG), chest width (CW), hip height (HH), hip width (HW), rump length (RL), rump width (RW), and thurl width (TW) which their details are illustrated in Fig. S1. Descriptive statistics for each trait are shown in Table 1.

### Genotypic data

Genotyping data used in genomic evaluations in this study were available for 1679 individuals that had been genotyped using Illumina BovineSNP50K (*n* = 959) and HD 777 K (*n* = 720) BeadChip (Illumina Inc., San Diego, CA, USA). From both the 50 K and 777 K SNP chips only the identical locations were used and 45,304 common SNPs were found. Animals with more than 10% of missing genotype data (*n* = 73) and without a phenotype for the traits of interest (*n* = 33) as well as animals with Mendelian conflicts (*n* = 11) or deviation errors between the pedigree and genomic relations (*n* = 39) were excluded from the final analyses. The Mendelian conflicts were investigated using all SNP to determine wrong relationships for sire-offspring pairs. The exclusion threshold of Mendelian conflicts was assumed two percentages according to the default of PreGSf90 program [49]. To detect deviation errors between the pedigree and genomic data, the relationship matrix based on pedigree (**A**) and SNP genotypes (**G**) were compared. A total of 39 individuals showed unreasonable deviations based on their **A** and **G** relationship coefficients possibly due to DNA sampling errors and thus were eliminated. Among these, duplicated individuals (*n* = 8) which might have been genotyped twice with different IDs had their **G** coefficients close to 1.0 and **A** coefficients close to 0 (*n* = 4) or 0.25 (*n* = 4). For the remaining individuals (*n* = 31), either the **G** coefficients were near 0 while the **A** coefficients were close to 0.25, in this case, they would have been mistakenly recorded as half-sib individuals, or the **G** coefficients were close to 0.25 and the **A** coefficients were near 0 as would be half-sibs mistakenly recorded as unrelated. SNPs with unknown positions (302 SNPs) and those located on sex chromosomes (1150 SNPs) were removed from the analyses after quality control. Furthermore, the SNPs with call rates lower than 0.98 (2677 SNPs), minor allele frequencies lower than 0.01 (6684 SNPs), and a maximum difference between observed and expected frequency of 0.15 as a departure of heterozygous from the Hardy-Weinberg equilibrium (31 SNPs) were excluded. The missing genotypes were imputed in the BEAGLE software [50]. Finally, the genotypes for 34,460 SNP markers from 1523 animals (369 bulls and 1154 steers) were used for the analyses.

### Statistical methods

#### Estimation of variance components

The variance components and heritabilities were estimated implementing AIREMLF90 software [49], using the pedigree-based single-trait animal model as follows:

1 $$ \mathbf{y}=\mathbf{Xb}+\mathbf{Zu}+\mathbf{e} $$

where **y** is the vector of the observations for the trait of interest, **b** is the vector of the fixed effects, including batch-test place-sex [the batch was formed twice every year and it represents the year and season of selection at 6 months of age and test place is the place where animals were reared after selection (164 levels)], birth place [the county where the farms were located (111 levels)], and age at the recorded date as a covariate; **u** is the vector of additive genetic effects of the individuals; **X** is the incidence matrix of **b**; **Z** is the incidence matrix of **u**, and **e** is the vector of the residuals. It was assumed that **u** ~ N (0, **A**σ_a_^2^) and **e** ~ N (0, **I**σ_e_^2^), where **A** was a pedigree-based genetic relationship matrix and σ_a_^2^ was the additive genetic variance, and σ_e_^2^ is the residual variance.

Finally, the adjusted phenotypes (y_adj_) were obtained for each trait and animal as the residual effects (**e**) of the **y** = **Xb + e** model which $\hat{\mathbf{b}}={\left({\mathbf{X}}^{\prime}\mathbf{X}\right)}^{-\mathbf{1}}\ {\mathbf{X}}^{\prime}\mathbf{y}$.

#### Estimation of breeding values

Four methods, a traditional BLUP method with phenotypes only on genotyped animals (PBLUP-G), a traditional BLUP method with pedigree-based relationship matrix (PBLUP), a GBLUP method based on genomic relationship matrix, and a single-trait single-step GBLUP (ssGBLUP) method by combining the relationship matrix constructed from genotyped and non-genotyped individuals and pedigree information, were used to predict breeding values.

#### Pedigree-based best linear unbiased prediction (PBLUP) model

The BLUP model to predict conventional EBV was:

2 $$ {\mathbf{y}}_{\mathrm{adj}}=\mathbf{1}\mu +\mathbf{Zu}+\mathbf{e} $$

where **y**_adj_ is the vector of the observations for the trait adjusted for fixed effects, **1** is the vector of ones; μ is overall mean; other notations are the same as in the model eq. (1). In addition, the EBVs were obtained using only phenotypes and pedigree on genotyped animals (PBLUP-G model).

#### Genomic best linear unbiased prediction (GBLUP) model

For estimating genomic breeding values, we used the model (2) above with the following assumptions;

**u** was the vector of additive genetic effects of only genotyped individuals and **Z** was the incidence matrix of **u**. It was assumed that **u** ~ N (0, **G**σ_a_^2^), where **G** was the genomic relationship matrix constructed using SNP information as described by VanRaden [15]:

$\mathbf{G}=\frac{\mathbf{M}{\mathbf{M}}^{\prime }}{2{\sum}_{\mathrm{i}=1}^{\mathrm{m}}{\mathrm{p}}_{\mathrm{i}}\ \left(1-{\mathrm{p}}_{\mathrm{i}}\right)}$, where m is the total number of markers (34,460), p_i_ is the allelic frequency of i^th^ marker and **M** is the matrix of centered genotypes.

#### Single-step genomic best linear unbiased prediction (ssGBLUP) model

In the ssGBLUP method, the statistical model was similar to that used for traditional evaluation; however, the non-genotyped and genotyped animals were simultaneously included in the hybrid relationship matrix of **H** that was a combination of **A** (numerator relationship matrix) and **G** (the genomic relationship matrix) matrices. The inverse of the **H** matrix was obtained as the following equation [51, 52] and by employing preGSf90 software [53]:

$$ {\mathbf{H}}^{-1}={\mathbf{A}}^{-1}+\left[\begin{array}{cc}\mathbf{0}& \mathbf{0}\\ {}\mathbf{0}& {\left(0.95\mathbf{G}+0.05{\mathbf{A}}_{\mathbf{22}}\right)}^{-1}-{\mathbf{A}}_{22}^{-1}\end{array}\right] $$

Where **A**_22_ is the numerator relationship matrix for genotyped animals.

#### Validation and prediction of accuracy

In this study, the accuracy and unbiasedness of prediction were obtained using five-fold cross-validation sets for all traits. Genotyped individuals were assigned to five mutually exclusive groups for cross-validation. K-means clustering, according to pedigree relationship coefficients, was used to minimize the relatedness between training and validation sets [54]. The five groups included 360, 356, 174, 466, and 167 individuals, respectively. Each group was used as the validation set, while the remaining genotyped individuals were included in the training set. When using the ssGBLUP and PBLUP methods with phenotypes of all animals, non-genotyped individuals were included in the training set. For each validation set, the prediction accuracy was calculated as the correlation between the vector of adjusted phenotypes and the vector of estimated breeding values, divided by the square root of trait heritability. Regressions of adjusted phenotype on the EBV and the root of mean square error (RMSE) were calculated for all prediction methods.

## Supplementary Information

**Additional file 1: Figure S1.** Location of body measurement parts in Hanwoo cattle.

## Data Availability

All data analyzed during this study are included in this published article and its supplementary information file.

## Abbreviations

HIC: Hanwoo Improvement Center
PBLUP: Pedigree best linear unbiased prediction
GBLUP: Genomic best linear unbiased prediction
ssGBLUP: Single-step genomic best linear unbiased prediction
QTL: Quantitative trait loci
SNP: Single nucleotide polymorphism
BH: Body height
BL: Body length
CD: Chest depth
CG: Chest girth
CW: Chest width
HH: Hip height
HW: Hip width
RL: Rump length
RW: Rump width
TW: Thurl width
GEBV: Genomic estimated breeding value
RMSE: Root of mean square error
