# Supplementary material for: Improving the accuracy of genomic evaluation for linear body measurement traits using single-step genomic best linear unbiased prediction in Hanwoo beef cattle
Source: BMC Genet. 2020 Dec 2;21:144. doi: 10.1186/s12863-020-00928-1 (PMC7709290; doi:10.1186/s12863-020-00928-1)
Supplement: Supplementary file 1 — Additional file 1: Figure S1. Location of body measurement parts in Hanwoo cattle. [file 12863_2020_928_MOESM1_ESM.docx]

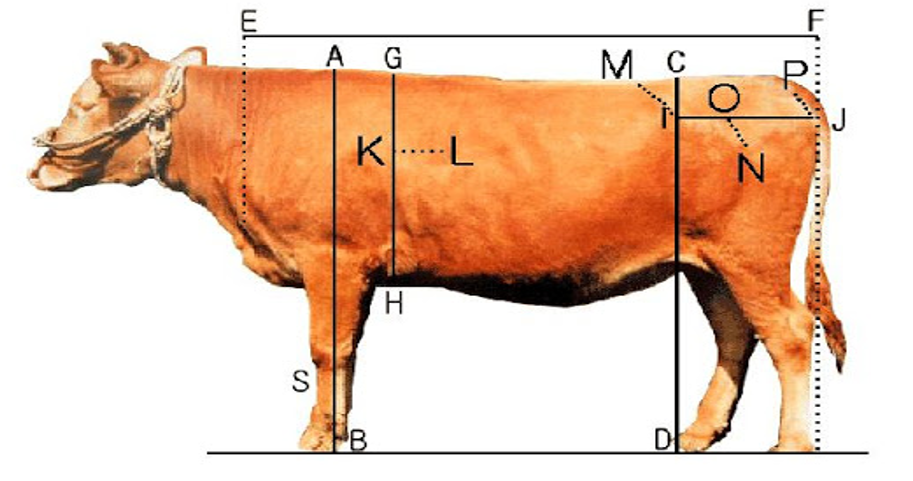


Figure S1. Location of body measurement parts in Hanwoo cattle.

Body height: A – B; Body length: E – F; Chest depth: G – H; Chest width: K – L; Hip height: C – D; Hip width: I – M; Rump length: I – J; Rump width: N – O; Thurl width: J – P.

Chest girth is measured using a tape along the same line as chest depth.
